# Chinese Medicine in Diabetic Peripheral Neuropathy: Experimental Research on Nerve Repair and Regeneration

**DOI:** 10.1155/2012/191632

**Published:** 2012-08-15

**Authors:** Yuanlin Piao, Xiaochun Liang

**Affiliations:** Department of Traditional Chinese Medicine, Peking Union Medical College Hospital, Peking Union Medical College and Chinese Academy of Medical Sciences, No. 1 Shuaifuyuan, Dongcheng District, Beijing 100730, China

## Abstract

Diabetic peripheral neuropathy (DPN) is one of the most common complications of chronic diabetes mellitus. Pathological characteristics of DPN include axonal atrophy, nerve demyelination, and delayed regeneration of peripheral sensory nerve fibers. The goal of treatment in DPN is not only to ameliorate neurological symptoms but also to slow or reverse the underlying neurodegenerative process. Schwann cells and neurotrophic factors play important roles in the repair and regeneration of peripheral nerves. The present paper reviews current studies and evidence regarding the neurological effects of traditional Chinese medicine, with an emphasis on recent developments in the area of nerve repair and regeneration in DPN.

## 1. Introduction

Diabetic peripheral neuropathy (DPN) is a common complication of chronic diabetes. Pathological characteristics of DPN include axonal atrophy, nerve demyelination, and delayed regeneration of peripheral sensory nerve fibers. To our knowledge, the pathophysiological mechanism of DPN in dysfunctional peripheral nerve repair and regeneration is not well understood. 

The symptoms associated with DPN have been mentioned in various traditional Chinese medicine (TCM) references. Pujifang (Prescriptions for Universal Relief), an ancient Chinese medicine book written in the Ming dynasty, described the following constellation of symptoms: “The kidney pattern of diabetes consists of symptoms of thirst, dry eye, impotence, and annoying pain in the hands and feet.” Moreover, in Wangxugaoyian (Medical Records of Wangxugao) from the Qing dynasty, there was a case of a patient with diabetes noted to have “numbness of hands and feet” and “limbs as cold as ice.” The differentiation of DPN implicates the domains of “sinew impediment,” “blood impediment,” and “leg flaccidity” in Chinese medicine [1].

From the viewpoint of TCM [1], the etiology and pathogenesis of DPN are as follows: (1) with an increased duration of disease in diabetes, a deficiency of yin burns body fluid and blood, resulting in empty heat. This increases blood viscosity, resulting in blood stasis, as well as blockage of sinews and channels; (2) excessive intake of foods high in fat and sugar content results in the deficiency of spleen and stomach, resulting in the accumulation of dampness and phlegm, which has a synergistic effect with stasis; (3) sinew and channels demonstrate poor nourishment because of the deficiency of liver and kidney; (4) the deficiency of yin results in a deficiency of yang, which generates an inner cold that results in microvascular coagulation. These four aspects result in a decreased peripheral flow of qi and blood to muscles, sinew, and channels. With regard to visceral organ systems, DPN is related to the liver, spleen, and kidney. The nature of DPN is deficiency secondarily complicated by excess; the deficiency is the root, and the excess is a subsequent manifestation. The root cause is deficiency in qi, yin, and yang; the subsequent complication is blood stasis and phlegm accumulation. Common patterns and treatments of DPN are summarized in Table 1.

Recently the effects of Schwann cells and neurotrophic factors on the repair and regeneration of peripheral nerve have been of research interest. Recent studies have shown that TCM medications may affect neuronal repair and regeneration in DPN. In this paper, we examine current experimental research in Chinese literature and discuss the possible mechanisms of action of TCM on DPN, focusing on its effects on Schwann cells and neurotrophic factors (Table 2).

The literature search was conducted in the following database: China Journals Full-Text Database (2002–2012) (http://dlib.cnki.net/kns50/index.aspx). The keywords used were: nerve repair, nerve regeneration, Chinese medicine, acupuncture, sciatic nerve, diabetic rats, Schwann cell, neurotrophic factors, and diabetic neuropathy. The authors read full articles and reached consensus after discussion. The effects and mechanisms of Chinese medicine on nerve repair and regeneration were reviewed. Articles included in the study covered the following domains of TCM: (1) Chinese herbal medicine therapy and (2) acupuncture and moxibustion. Research of monomers, review articles, and abstracts were excluded. A total of 21 peer-reviewed papers written in Chinese were included in this paper.

## 2. Schwann Cells

Schwann cells are glial cells of the peripheral nerve system. They are important for maintaining the microenvironment for regeneration of peripheral nerves. Schwann cells not only support the repair of peripheral nerves, but they also induce, stimulate, and modulate axonal regeneration and myelin formation via expression and secretion of multiple proteins, peptides, and other bioactive substances. Thus, Schwann cells play an important role in promoting repair and regeneration after peripheral nerve injury. In hyperglycemia, a series of changes, including abnormal expression of proteins and enzymes, result in increased apoptosis and decreased cell proliferation and repair signals [23, 24]. Therefore, inhibiting apoptosis and promoting growth of Schwann cells may be crucial in the prevention and treatment of DPN.

### 2.1. Chinese Medicine Promotes Schwann Cell Proliferation

**Figure 1:**
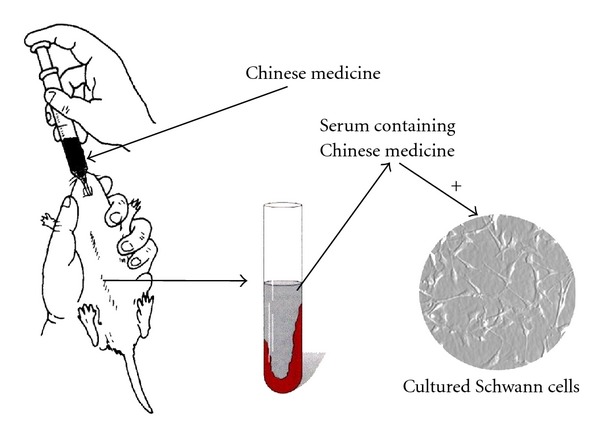
Preparation of serum containing Chinese medicine.

Multiple studies have demonstrated the presence of axonal degeneration and peripheral nerve demyelination in DPN. Characteristic histopathological findings include lipid droplets, Reich granules, and glycogen granules in the cytoplasm of Schwann cells, mitochondrial swelling, and disappearance of mitochondrial cristae, which are indicative of a proapoptotic state. Schwann cell proliferation and migration promote nerve regeneration and thus are likely to mitigate in DPN. Sun et al. [2] applied the serum pharmacological method (Figure 1) to investigate the effect of serum containing Jinmaitong on the proliferation of Schwann cells cultured under hyperglycemic conditions. Compared with a control group treated with neurotrophin, there was no significant difference between the two groups in their effect on enhancing the proliferation of Schwann cells. Furthermore, both groups also increased the expression of nerve growth factor (NGF) in the same cultured Schwann cells. Wu et al. [3] used the XTT method and the 3H2TdR incorporation assay to assess the activity and proliferation of Schwann cells isolated from sciatic nerve tissues of newborn Wistar rats. They confirmed that allyl glycosides significantly reversed the inhibition of proliferation of Schwann cells induced by hyperglycemia.

### 2.2. Chinese Medicine Inhibits Apoptosis of Schwann Cells

Apoptosis is a manifestation of cell damage. The typical histopathological pattern of mitochondrial swelling and dissolved mitochondrial cristae occur in response to streptozotocin (STZ) administered to diabetic rats [25]. *In vitro* experiments showed that hyperglycemia reproduces this pattern of apoptosis in Schwann cells [26]. Ji et al. [4] reported that application of Jiangtangshuluofang normalized levels of serum insulin and glycosylated hemoglobin and inhibited the apoptosis of Schwann cells surrounding sciatic nerves in diabetic rats. The mechanism is thought to be related to the inhibition of proapoptotic factors caspase-3 and Bax and promoting of expression of Bcl-2, an antiapoptotic factor. Liu et al. [5] reported that the Chinese medicines astragalus, salvia, and yam have antiapoptotic actions on Schwann cells cultured under hyperglycemic conditions. These medicines increased levels of Bcl-2 expression, while inhibiting expression of caspase-3. Furthermore, the combination of those three herbs was synergistic. In vitro studies showed that the medicated serum containing Jinmaitong decreased the expression of inducible nitric oxide synthase (iNOS), NADPH oxidase p22-phox, 8-OHdG, and active caspase-3 (17 kDA) in Schwann cells, suggesting that Jinmaitong can reduce oxidative injury and apoptosis associated with hyperglycemic conditions (Table 3) [6, 7]. 

## 3. Chinese Medicine's Effects on Neurotrophic Factors

Neurotrophic factors are essential for the maintenance and survival of neurons. When peripheral nerve are injured, neurotrophic factors can bond to specific tyrosine kinase receptors on the surface of target cells, preventing neuronal cell death and promoting the repair of neurons and axon regeneration. Neurotrophic factors can be classified into: neurotrophins (including NGF, brain-derived neurotrophic factor, and neurotrophin); neuropoietic cytokines (including ciliary neurotrophic factor [CNTF] and interleukins); and the transforming growth factor-beta (TGF-*β*) superfamily (which can be subdivided into acidic fibroblast growth factors and basic fibroblast growth factors). In addition, there are other neurotrophic factors, such as insulinlike growth factor (IGF) and glial-derived neurotrophic factor [27]. Present studies suggest that diabetes-induced dysfunction of nerve regeneration results partially from decreased levels of some neurotrophic factors or their receptors.

### 3.1. NGF

NGF was the first discovered and most typical neurotrophic factor. It plays an important role in neuronal development, differentiation, and the maintenance of normal functions. NGF not only protects neurons and reduces their degeneration and death, but it also promotes nerve regeneration after nerve injury. There is a deficiency of NGF in diabetes, and reduced levels or activity of NGF plays a significant role in the pathogenesis of diabetic neuropathy [28]. Qu et al. [8] observed that 12 weeks after the success of a STZ-induced diabetic neuropathy rat model, compared with the normal rats, the tail-flick latency was significantly prolonged, the pain threshold was significantly lower, and NGF protein and mRNA expression in the sciatic nerve were significantly reduced in the model rats. Moreover, NGF-mRNA expression level in the sciatic nerve was negatively correlated with the tail-flick latency and was positively correlated with the mechanical pain threshold in the model rats. The model rats were orally administered with the Chinese medicine compound Jinmaitong in three different dosage groups: large, medium, and small. After the intervention, the medium-dosage group of Jinmaitong showed that the tail-flick latency was significantly reduced, the pain threshold significantly increased, and sciatic NGF-mRNA and protein expression were significantly increased, compared with the model control group. Furthermore, in vitro study proved that the serum containing Jinmaitong promotes secretion of NGF in high-glucose cultured Schwann cells [2]. Deng and Zhang [9] investigated the effects of Qitengtongluoyin on protein expression of NGF and neuropeptide substance P (SP) in sciatic nerves of STZ-induced diabetic multiple neuropathy rats, and proved that Qitengtongluoyin can prevent and treat sciatic neuropathy in STZ-induced diabetic multiple neuropathy rats, probably via promoting the expression of NGF and SP protein. Yu et al. [10] reported that a 6-week intervention of the Chinese medicine compound Tangmoning in STZ-induced diabetic rats resulted in a significant increase of NGF mRNA and that the effect was similar to that of methycobal. Xu and Yang [11] reported that Yishentongluofazufang can increase the NGF content in the serum of STZ-induced rats. Wang and Liu [12] proved that Tangbikang can increase serum NGF level and increase the expression of NGF mRNA of sciatic nerve in diabetic rats. Ma et al. [13] testified in their experimental study that certain extracts of morus alba can improve diabetic peripheral neuropathy in alloxan-induced diabetic rats, via promoting expression of NGF and myelin protein in sciatic nerves. 

Besides the above-mentioned Chinese herbal medicine studies, recent studies have shown that acupuncture and moxibustion can improve DPN, possibly via their effects on NGF. Dong et al. [14] conducted electroacupuncture (EA) on the points of *Shenshu* (BL 23) and *Zusanli* (ST 36) in STZ-induced diabetic rats. After the intervention was done 12 times, the EA group showed increased NGF-positive cells and increased NGF mRNA expression in sciatic nerve, compared with the model group, suggesting EA upregulates expression of protein and mRNA of NGF and improves nerve repair in DPN. Huang et al. [15] found that EA treatment of diabetic rats resulted in increased expression of NGF mRNA in the sciatic nerve. Yin et al. [16] applied moxibustion on STZ-induced diabetic rats, moxaed at the points of *Yishu* (Ex-B3) and *Zusanli* (ST 36), 15 min each point, once daily for 56 consecutive days, found that blood glucose significantly decreased, nerve conduction velocity significantly increased, and NGF content significantly increased in treatment group, compared with those in model group, suggesting that moxibustion has functions of peripheral nerve protection which may be related to its promotion of NGF expression of nerve. 

### 3.2. CNTF

CNTF has multiple biological activities: promoting survival of neurons and protecting motor neurons; inhibiting degeneration of axons of motor nerves; enhancing growth speed of axon; preventing muscle atrophy. In addition, administration of CNTF results in promoting regeneration of peripheral nerves [29]. CNTF protein and bioactivity are reduced in the peripheral nerve of diabetic rats, and CNTF treatment improved nerve regeneration and prevented nerve-conduction slowing in diabetic rats, suggesting CNTF plays an important role in nerve regeneration in DPN [30]. Wang et al. [17] observed the Chinese medicine compound Jinmaitong's effects on CNTF expression in diabetic neuropathy rats, and confirmed that Jinmaitong can upregulate the expression of the protein and mRNA of CNTF in the sciatic nerves of diabetic neuropathy rats. For in vitro study, Wang et al. [18] prepared drug-containing serums with the application of 15 times the adult dosage of both Jinmaitong and neurotrophin and grouped as the blank control group (no cells added), normal control group (added with normal rat serum), high-glucose group (added with glucose), Jinmaitong group (added with serum containing Jinmaitong and normal rat serum), and neurotropin group (added with serum containing neurotropin). Except for the blank control group and normal control group, the 50 mmol/L glucose was added to all the groups to achieve high-glucose Schwann cell models. The expression of CNTF and CNTF mRNA was detected by SABC immunohistochemistry method and real-time fluorogenetic quantitative PCR, respectively. Results showed that compared with the normal control group, the CNTF and CNTF mRNA expression in the high-glucose group, Jinmaitong group, and neurotropin group were lowered. Compared with the high-glucose group, the CNTF and CNTF mRNA expression of the Jinmaitong group and neurotropin group were increased, and the CNTF mRNA expression in the Jinmaitong group was higher than that in the neurotropin group. This suggests that Jinmaitong upregulates the expression of CNTF and CNTF mRNA of Schwann cells cultured in high-glucose medium.

### 3.3. IGF-1

IGF-1 promotes cell growth and proliferation, and it promotes the growth of axons. Recent studies have shown that IGF-1 nourishes and supports motor and autonomic nerves. Decreased serum IGF-1 level and IGF-1 mRNA expression are shown in experimental diabetic rats, and administration of IGF-1 results in improvement of diabetic neuropathy [31]. Zeng et al. [19] applied reverse transcription polymerase chain reaction assay and confirmed that the expression of IGF-1 mRNA was decreased in sciatic nerves in STZ-induced diabetic rats; the expression level of IGF-1 mRNA and glucose was negatively correlated; treatment with Xiaokelingnongsuoye resulted in increased expression of IGF-1 mRNA in sciatic nerves. Xu et al. [20] set up a rat model of diabetic peripheral neuropathy and investigated Qingyingtang's effects on sciatic nerve conduction velocity, histopathological changes, and the expression of IGF-1 in tissue. They found that Qingyingtang enhanced sciatic nerve conduction velocity, improved histopathological changes, and increased the expression of IGF-1 in serum and tissue, confirming that Qingyingtang nourishes the sciatic nerve and promotes the sciatic nerve repair, possibly via increasing expression of IGF-1. Another study reported that Tangmoning can improve pathological changes in the sciatic nerve in rats, and it has protective effects on DPN, which might be related to its upregulation of the expression of IGF-1 protein [21]. Yin et al. [16] proved that moxibustion can upregulate IGF-1 mRNA expression of sciatic nerve in diabetic rats. 

### 3.4. Interleukin

Interleukin 1 (IL-1) plays a central role in the regulation of immune and inflammatory responses. It promotes cell proliferation and generation of other cytokines, and regulates metabolism. Recent studies found that IL-1 and IL-6 have functions of promoting regeneration of peripheral nerves; Schwann cell can secret IL-1 which promotes Schwann cells' secretion of NGF; IL-6 can promote nerve regeneration of via up-regulating the expression of CNTF mRNA [32–34]. It has not been reported that Chinese medicine promotes nerve repair and regeneration via interleukin. Only a few studies reported that Chinese medicine inhibited inflammation factors in diabetes, so that improved the nerve impairment in DPN. Zhang et al. [22] administered different dosages of Xiaoketongbi to STZ-induced diabetic rats for 2 months and found that Xiaoketongbi improved the peripheral neuropathy and decreased the levels of IL-1*β*, TNF-*α*, and CD54 in diabetic peripheral neuropathy rats, suggesting Xiaoketongbi relieves and improves diabetic neuropathy by means of inhibition of inflammation in diabetes. 

## 4. Conclusion

**Figure 2:**
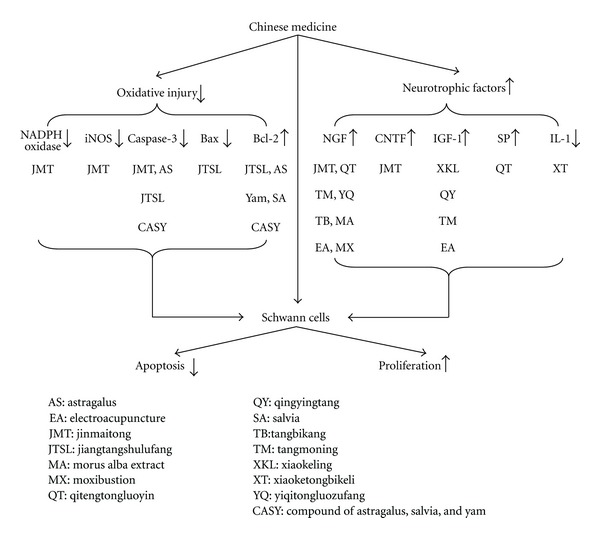
Chinese medicine's effects on Schwann cells and neurotrophic factors and its possible mechanisms of promoting nerve repair and regeneration in diabetic peripheral neuropathy.

Diabetic peripheral neuropathy is the result of multiple factors, and the repair and regeneration of peripheral nerves are very complicated procedures that are regulated by multiple factors; furthermore, the micro-environment that is needed for nerve repair and regeneration is not of single factor but composed of multiple related factors. Recent studies have shown that Chinese medicine inhibits apoptosis, promotes proliferation in Schwann cells, and increases expression of multiple neurotrophic factors; therefore, Chinese medicine can improve nerve repair and regeneration in DPN (Figure 2). Chinese medicine has the advantage of providing multiple therapeutic effects on multiple targets, compared with Western medicine, which uses conventional chemical agents and focuses on a single target. Therefore, to a certain extent, the effective single herb or compound of Chinese medicine might offer a more suitable micro-environment, one that is neurologically and physically needed for promoting repair and regeneration of nerves.

As we discussed in this paper, some studies investigated a single factor, thus, limited in explanations of the mechanisms of Chinese medicine's effects on nerve repair and regeneration. Although some Chinese medicine showed effectiveness in vitro studies, it might not be effective clinically, because in vitro cultured cells independently survive in an artificial environment, which is very different from the environment *in vivo*. Because of the diversity of patterns of Chinese medicine, uncertainty about activities of various ingredients, difficulty of quality control, and unknown interactions between components in the same Chinese medicine compound, research on the mechanisms is very difficult to get further. In short, further research is needed to clarify Chinese medicine's clinical value and the mechanisms of Chinese medicine's functions of nerve repair and regeneration in DPN. Future studies should be carried out with emphasis on both prevention and treatments to clarify the mechanisms by which Chinese medicine promotes nerve repair and regeneration; in the meantime, we need to explore and block the factors that inhibit nerve repair and regeneration.

## Figures and Tables

**Table 1:** Common patterns and treatments of DPN in Chinese medicine.

Pattern	Clinical manifestation	Therapeutic principle	Treatment (formula)	Origin of formula	Components
Deficiency of Yin and blood stasis	Numbness and burning pain in hands and feet gradually extends to the entire limbs, aggravated at night; night sweating; spontaneous sweating; heat sensation in the chest, palms and soles; soreness and weakness of the lumbar region and knees joints, dry mouth; thirsty; dry stool; dark-red tongue with peeled or little coating; fine and choppy pulse.	Nourish Yin and remove blood stasis	Zhibai Dihuang Wan 1 (Anemarrhena-Phellodendron-Rehmannia Pill) together with Taohongsiwutang 2 (Persica-Carthamus Four Substances Decoction)	1 Jinguiyaolue (Synopsis of Prescriptions of the Golden Chamber); 2 Yizongjinjian (Golden Mirror of Medicine)	*Shudihuang* (Radix Rehmanniae preparata), *Shanzhuyu* (Fructus Corni), *Shanyao* (Rhizoma Dioscoreae), *Zexie* (Rhizoma Alismatis), *Fuling* (Poria), *Mudanpi* (Cortex Moutan), *Zhimu *(Rhizoma Anemarrhenae), *Huangbai* (Cortex Phellodendri), *Danggui *(Radix Angelicae sinensis), *Chuanxiong* (Rhizoma Chuanxiong), *Baishao* (Radix Paeoniae alba), *Taoren* (Semen Persicae), *Honghua* (Flos Carthami tinctorii)

Deficiency of Yang and blood stasis	Numbness pain, aggravated at night or encounters coldness, aversion to cold, cold limbs, soreness and weakness of the lumbar region and knees joints, tastelessness in the mouth without thirsty, impotence, premature ejaculation, poor or loose stool, pale or dark tongue, white thick or greasy coating, deep fine or deep slow pulse.	Warm and tonify Yang and remove blood stasis	Jinguishenqiwan1 (Golden Chest Kidney-Qi Pill) together with Taohongsiwutang 2 (Persica-Carthamus Four Substances Decoction)	1 Jinguiyaolue (Synopsis of Prescriptions of the Golden Chamber); 2 Yizongjinjian (Golden Mirror of Medicine)	*Fuzi *(Radix Aconiti lateralis preparata), *Guizhi* (Ramulus Cinnamomi), *Shanzhuyu *(Fructus Corni), *Shanyao *(Rhizoma Dioscoreae), *Zexie *(Rhizoma Alismatis), *Mudanpi *(Cortex Moutan), *Fuling* (Poria), *Shudihuang* (Radix Rehmanniae preparata), *Danggui* (Radix Angelicae sinensis), *Chuanxiong* (Rhizoma Chuanxiong), *Baishao* (Radix Paeoniae Alba), *Taoren* (Semen Persicae), *Honghua* (Flos Carthami tinctorii)

Deficiency of Yin turning into wind	Numbness or soreness in hands and feet, dizziness, and vertigo and a feeling of falling, soreness, and weakness of the lumbar region and knee joints, heat sensation in the chest, palms and soles; staggering gait, red tongue with peeled coating, deep fine, and wiry pulse.	Nourish Yin and extinguish wind	Yiguanjian 1 (One Linking Decoction) together with Zhenganxifengtang 2 (Pacifying the Liver and Extinguishing Wind Decoction)	1 Liuzhouyihua (Medical Talks of Liuzhou); 2 Yixuezhongzhongcanxilu (Medical Records Loyal to Chinese Medicine and Reference to Western Medicine)	*Shashen* (Radix Glehniae), *Maidong* (Radix Ophiopogonis), *Danggui* (Radix Angelicae sinensis), *Shengdihuang* (Radix Rehmanniae), *Gouqi *(Fructus Lycii chinensis), *Niuxi *(Radix Achyranthis bidentatae), *Daizheshi *(Haematitum) *Longgu *(Mastodi Ossis fossilia), *Muli* (Concha Ostreae), *Guiban* (Plastrum Testudinis), *Xuanshen* (Radix Scrophulariae), *Tianmendong *(Radix Asparagi), *Baishao *(Radix Paeoniae alba), *Yinchenhao* (Herba Artemisiae scopariae), *Chuanlianzi *(Fructus Toosendan),* Maiya *(Fructus Hordei germinatus), *Gancao* (Radix Glycyrrhizae uralensis)

Phlegm and blood stasis blocking the channels	Numbness, paresthesia, aversion to cold or heat, soreness and weakness of the lumbar region and knees joints, a feeling of heaviness in lower limbs, muscle atrophy, epigastric fullness, loss of appetite, poor or loose stool, nocturnal emission, impotence or premature ejaculation, swollen tongue with thick greasy coating, deep-fine or deep-choppy pulse.	Dispel phlegm and remove blood stasis	Pishenshuangbuwan1 (Spleen-Kidney Double Supplement Pill), Erchentang 2 (Two Old Decoction) and Taohongsiwutang 3 (Persica-Carthamus Four Substances Decoction)	1 Xianxingzhaiyixueguangbiji (Extensive Medical Notes of Early Awake House); 2 Taipinghuiminhejijufang (Prescriptions from the Great Peace Imperial Grace Pharmacy); 3 Leizhengzhicai (Classified Patterns with Clear-cut Treatments)	*Renshen* (Radix Ginseng), *Lianzi* (Semen Nelumbinis), *Tusizi* (Semen Cuscutae), *Wuweizi *(Fructus Schisandrae), *Shanzhuyu* (Fructus Corni), Shanyao (Rhizoma Dioscoreae), *Cheqianzi *(Semen Plantaginis), *Roudoukou* (Semen Myristicae), *Juhong *(Exocarpium Citri rubrum), Sharen (Fructus Amomi), *Bajitian* (Radix Morindae officinalis), *Buguzhi* (Fructus Psoraleae), *Banxia* (Rhizoma Pinelliae preparatum), *Chenpi* (Pericarpium Citri reticulatae), *Fuling *(Poria), *Zhigancao* (Radix Glycyrrhizae preparata), *Shudihuang* (Radix Rehmanniae preparata), *Danggui* (Radix Angelicae sinensis), *Chuanxiong* (Rhizoma Chuanxiong), *Baishao* (Radix Paeoniae alba), *Taoren *(Semen Persicae), *Honghua* (Flos Carthami tinctorii)

**Table 2:** Key data from cited studies in Chinese literature.

First author (year) ref.	Tissue/cells	Experimental treatment	Control treatment	Methods for main indicators	Main outcomes	Author's conclusion
Sun (2009) [2]	Schwann cells	Serum containing Jinmaitong (Sinew-Channel Unobstruction)	Neurotropin	MTT assay for proliferation of Schwann cells	Promoted proliferation of Schwann cells and increased expression of NGF of Schwann cells cultured in high glucose	“Can promote the proliferation of Schwann cells and increase the expression of NGF of Schwann cells cultured in high glucose”

Wu (2009) [3]	Schwann cells	Allyl glycoside extracted from Herba Rhodiolae	Shenmaizhusheye (Ginseng-Ophiopogonis injection)	XTT method and 3H-TdR incorporative method for activity and proliferative capability of Schwann cells, respectively	Improved proliferative capability of Schwann cells	“Can improve inhibitory effect on proliferative capability of Schwann cell in high glucose milieu, *in vitro*”

Ji (2009) [4]	Sciatic nerve	Jiangtangshuluofang (Decrease Glucose Free Collaterals Prescription) for 8 W	Methycobal and Gliclazide	Radioimmunoassay for insulin level and HbA1c; tunel for apoptosis; immunohistochemical method for expression of Bcl-2, Bax, and caspase-3	Increased Bcl-2 expression; reduced caspase-3 and Bax expression; decreased apoptosis of Schwann cells.	“Can increase the insulin level and lower HbA1c level in diabetic rats, inhibit Schwann cells apoptosis; the mechanism might be related to its inhibition of caspase-3 and Bax expression, and promoting the expression of Bcl-2”

Liu (2010) [5]	Schwann cells	Huangqi (Radix Astragali), Danshen (Radix Salviae miltiorrhizae), Shanyao (Rhizoma Dioscoreae), and compounds of three herbal medicines	None	Flowcytometer for apoptosis rate of Schwann cells; real-time PCR for expression of Bcl-2 and caspase-3 mRNA; Western blotting for expression of Bcl-2 and caspase-3 protein	Decreased apoptosis rate; increased Bcl-2 mRNA and protein expression; decreased caspase-3 mRNA expression in astragalus, salvia and compound groups; decreased expression of caspase-3 protein in astragalus, yam, and compound groups.	“The apoptosis of Schwann cell co-cultured with endothelial cell in high glucose can be protected by Chinese herbs, different herbs have different effect, the compound intervention group was the best”

Zhao (2011) [6]	Schwann cells	Serum containing Jinmaitong (Sinew-Channel Unobstruction)	Vitamin C	Immunofluorescence method for the expression of iNOS; real-time fluorescence quantitative PCR for p22-phox mRNA expression	Deceased expression of iNOS and p22-phox mRNA of Schwann cells	“Can down-regulate the expression of iNOS protein of NADPH oxidative p22-phox subunit mRNA of Schwann cells cultured in higher glucose medium”

Piao (2011) [7]	Schwann cells	Serum containing Jinmaitong (Sinew-Channel Unobstruction)	Vitamin C	Enzyme-linked immunoabsorbant assay for 8-OHdG level; immunofluorescence for expression of caspase-3 protein; real-time fluorescence quantitative PCR for expression of caspase-3 mRNA	Decreased 8-OHdG level; decreased expression of caspase-3 protein and mRNA in the supernatant of Schwann cells	“Can improve high-glucose induced oxidative injury of DNA apoptosis in Schwann cells, suggesting it might improve oxidative injury and apoptosis in diabetic neuropathy”

Qu (2008) [8]	Sciatic nerve	Jinmaitong (Sinew-Channel Unobstruction) for 16 W	Neurotropin	Real-time fluorescence quantitative PCR for NGF mRNA of sciatic nerve; immunohistochemical method for NGF protein of sciatic nerve	Increased NGF and NGF mRNA in sciatic nerve.	“Can upregulate the expression of NGF protein and NGF mRNA in sciatic nerve of rats with DPN”

Deng (2007) [9]	Sciatic nerve	Qitengtongluoyin (Astragalus Vine Free Collaterals Decoction) for 6 W	None	Immunohistochemical assay for expression of NGF and substance P in sciatic nerve	Improved changes of sciatic nerve, increased expression of NGF and substance P in sciatic nerve	“Has the preventive and treatment effects on pathological changes of sciatic nerve of DPN rats. It's possible mechanism may be associated with the promotion of expression of endogenous NGF and substance P”

Yu (2004) [10]	Sciatic nerve	Tangmoning (Glucose End Peace Granules) for 6 W	Methycobal	Reverse transcription PCR for NGF mRNA of sciatic nerve	Increased expression of NGF mRNA	“Appears to upregulate the expression of NGF mRNA of sciatic nerve in diabetic rats”

Xu (2009) [11]	Sciatic nerve, serum	Yiqitongluofazufang (Tonify Qi Free Collaterals Prescription) for 6 W	Methycobal	ELISA for serum NGF	Increased NO, decreased MDA, and increased NGF; improved histopathological changes	“Can prevent DPN via improvement of MDA, NO, and NGF”

Wang (2010) [12]	Sciatic nerve	Tangbikang (Sugar Blockage Recovery) for 8 W	Methycobal	Real-time fluorescence quantitative PCR for NGF mRNA of sciatic nerve; ELISA for NGF level in serum	Increased NGF level in serum and increased NGF mRNA expression.	“Can increase the NGF protein and mRNA expression to play an important role of peripheral nerve protection”

Ma (2007) [13]	Sciatic nerve	Morus alba extract for 8 W	Methycobal	Pathological observation for expression of NGF and myelin basic protein	Increased expression of NGF and myelin basic protein in sciatic nerve	“Morus alba extract has functions of increasing the expression of NGF and myelin basic protein thus improving the DPN”

Dong (2007) [14]	Sciatic nerve	*Shenshu* (BL 23), *Zusanli *(ST 36); G6805-II electroacupuncture device, continuous wave, frequency 2 Hz, 20 min every other day, for 12 times	Methycobal	Immunity and fluorescent quantitation PCR for NGF mRNA, and immunohistochemical staining analysis for NGF protein-positive cells of sciatic nerve	Increased expression of NGF protein and NGF mRNA in sciatic nerve.	“Can upregulate the expression of NGF protein and NGF mRNA, and promote sciatic nerve repair in DPN rats”

Huang (2010) [15]	Sciatic nerve	Positive electrode located 1.0 cm above the interior of proximal greater trochanter, distal fibular head and the inside of each 0.3 cm. Needle 0.5 cm in depth, with the stimulus intensity of 2-3 V, at the frequency of 100 beats/min, 45 min each day	None	Reverse transcriptase-PCR for expression of NGF mRNA and IGF-1 mRNA of sciatic nerve	From 2nd week, the expression of NGF mRNA and IGF-1 mRNA increased, at 10th week; the expression of NGF mRNA and IGF-1 mRNA sustained in higher level.	“Can elevate the mRNA expression of NGF and IGF-1 in sciatic nerve of diabetic rat, which may be one of the mechanisms of acupuncture on diabetic neuropathy”

Yin (2008) [16]	Sciatic nerve	Moxaed at the points of *Yishu* (Ex-B3) and *Zusanli* (ST 36), 15 min each point, once daily for 56 consecutive days	None	Neuroelectrophysiological detection for SNCV; ELISA for NGF of sciatic nerve	Decreased blood glucose level; increased SNCV; increased NGF content in sciatic nerve.	“The improving effect of moxibustion on diabetic peripheral neurological symptoms in a rat model of DPN may be related to an increase in the NGF content and promotion of peripheral neuroprotection”

Wang (2010) [17]	Sciatic nerve	Jinmaitong (Sinew-Channel Unobstruction) for 8 W	Neurotropin	The hydrothermal tail-flick and pain threshold to mechanical stimulation; SABC immunohistochemistry method for CNTF expression and real-time fluorescence quantitative PCR for CNTF mRNA expression in sciatic nerve	The pain thresholds were raised and tail-flick latencies were shortened; the expression of CNTF mRNA and protein was increased	“Can obviously upregulate the expression of CNTF mRNA and protein in the sciatic nerve of rats with neuropathy”

Wang (2010) [18]	Schwann cells	Serum containing Jinmaitong (Sinew-Channel Unobstruction)	Neurotropin	SABC immunohistochemistry method for CNTF expression and real-time fluorescence quantitative PCR for CNTF mRNA expression in Schwann cells	Increased CNTF and CNTF mRNA expression in Schwann cells	“Can upregulate the expression of CNTF and CNTF mRNA of rat Schwann cells in cultured high glucose medium, so as to improve DPN”

Zeng (2005) [19]	Sciatic nerve	Xiaokelingnongsuoye (Diabetes Agility Concentration Fluid) for 8 W	Methycobal	Relative quantity PCR for IGF mRNA	Increased expression of IGF-1 mRNA in sciatic nerve	“Is involved in the regulation of IGF-1 mRNA expression, and probably prevents diabetic peripheral neuropathy from deterioration”

Xu (2009) [20]	Sciatic nerve	Qingyingtang (Clearing Nutritive Qi Decoction) for 10 W	Methycobal	ELISA for IGF-1 in serum; IGF-1 in sciatic nerve and liver	Increased IGF-1 level in serum, liver and sciatic nerve	“Increase the expression of IGF-1 in tissue, and have effects of nerve repair in DNP rats”

Wang (2010) [21]	Sciatic nerve	Tangmoning (Glucose End Peace Granules) for 8 W	None	Western blotting for expression of NGF and IGF-1 in sciatic nerve; sciatic ultrastructure observation by transmission electron microscope	Increased expression of NGF and IGF-1; pathological changes of sciatic nerve were improved by transmission electron microscope	“Has some protective effect on sciatic nerve in diabetic rats. The mechanism may be related to the upregulation of the expression of NGF and IGF-1 proteins.”

Zhang (2008) [22]	Serum	Xiaoketongbikeli (Diabetes Free Obstruction Granula)	Methycobal	Radioimmunoassay for IL-1*β* and TNF-*α*, ELISA for CD54	Reduced the level of IL-1*β*, TNF-*α*, and CD54	“Can relieve or improve diabetic peripheral nerve injury by interfering with inflammation factors in diabetes”

MTT: methyl thiazolyl tetrazolium; ELISA: enzyme-linked immunoabsorbent assay; MDA: malondialdehyde; DPN: diabetic peripheral neuropathy; STZ: streptozotocin; NGF: nerve growth factor; ALL: Alloxan; PCR: polymerase chain reaction; CNTF: ciliary neurotrophic factor; SNCV: sensory nerve conduction velocity.

**Table 3:** Summary of formulas cited in this review.

First author(year) ref.	Formula	Traditional source of formula	Traditional indication	Components	Function	Rationale of formula
Ji (2009) [4]	Jiangtangshuluofang (Decrease Glucose Free Collaterals Prescription)	Empirical formula of Dr. Dashun Chen	DPN, pattern of deficiency of qi and yin, fluid deficiency heat and blood stasis, deficiency of blood turning into wind	*Shengdihuang* (Radix Rehmanniae), *Shanzhuyu* (Fructus Corni), *Gouqizi* (Fructus Lycii), *Gegen* (Radix Puerariae), *Huangqi* (Radix Astragali), *Danshen* (Radix Salviae miltiorrhizae), *Puhuang* (Pollen Typhae), *Shuizhi* (Hirudo), *Huanglian* (Rhizoma Coptidis), *Chantui* (Periostracum Cicadae), *Jili* (Fructus Tribuli)	Nourish yin tonify qi, clear heat and moisten dryness, activate blood and remove stasis, dispel wind and free collaterals.	Chief: *shengdihuang, shanzhuyu, gouqi zi* and *gegen* regender fluid and clear heat, nourish yin of liver, kidney and lung. Deputy: *huangqi* tonifies qi; *danshen, puhuang* and *shuizhi* remove stasis and free collaterals. Assistant: *huanglian* clears heat; *chantui* and *jili* relieve itching.

Piao (2011) [7]	Jinmaitong (Sinew-Channel Unobstruction)	Empirical formula of Dr. Xiaochun Liang	DPN, pattern of kidney deficiency and blood stasis; pattern of deficiency of both yin and yang, interior cold and stasis	*Tusizi* (Semen Cuscutae), *Nuzhenzi* (Fructus Ligustri lucidi), *Shuizhi* (Hirudo), *Yanhusuo* (Rhizoma Corydalis), Huangqi (Radix Astragali), Shengdihuang (Radix Rehmanniae), *Guizhi* (Ramulus Cinnamomi), *Xixin* (Herba Asari)	Tonify kidney, activate blood, warm and unblock channels and vessels.	Chief: *tusizi* tonifies yang and nourishes yin of kidney, secures essence and improves vision, and checks diarrhea; *nuzhenzi* nourishes yin of liver and kidney, and clears empty-heat. Deputy: *shuizhi* and *yanhusuo* break blood, expel stasis and relieve pain. Assistant: *huangqi* and *shengdihuang* tonify qi and nourish yin; *guizhi* and *xixin* warm and unblock channels and vessels, and promote qi and blood circulation.

Deng (2007) [9]	Qitengtongluoyin (Astragalus Vine Free Collatrals Decoction)	Empirical formula	DPN, pattern of qi deficiency and blood stasis	*Huangqi* (Radix Astragali), *Huangbai* (Cortex Phellodendri), *Niuxi* (Radix Achyranthis bidentatae), *Jixueteng* (Caulis Spatholobi), *Cangzhu* (Rhizoma Atractylodis), *Yiyiren* (Semen Coicis), *Qingdai* (Indigo Naturalis)	Tonify qi, activate blood and remove stasis.	Not mentioned about traditional rationale. The authors stated that **“**Based on modern pharmacological research. *Huangqi* strengthen immunity; *jixueteng* and *niuxi* modulate micro-circulation; *huangbai, cangzhu, niuxi* and *yiyiren* have functions of regulating glucose.”

Xu (2009) [11]	Yiqitongluofazufang (Tonify Qi Free Collaterals Prescription)	Empirical formula	DPN, pattern of kidney deficiency, qi deficiency and obstruction of collateral vessels.	*Shudihuang* (Radix Rehmanniae praeparata)*, Heshouwu* (Radix Polygoni multiflori)*, Luoshiteng* (Caulis Trachelospermi)*, Huangqi* (Radix Astragali), *Dilong* (Lumbricus)*, Wugong* (Scolopendra)*, Quanxie* (Scorpio)	Nourish yin, tonify kidney and free collaterals	Chief: *shudihuang* and *heshouwu* nourish yin and tonify kidney. Deputy: *luoshiteng* and *huangqi* tonify qi and free collaterals. Assistant: *dilong, wugong* and *quanxie* free collaterals and relieve pain.

Wang (2010) [12]	Tangbikang (Sugar Blockage Recovery)	A variation of Huangqiguizhi-Wuwutang (Astragalus-Cinnamomum Five-Ingredient Decoction)^∗^	Blood impediment, exhibiting numbness sensation in skin and limbs, slightly choppy and tight pulse.	*Huangqi* (Radix Astragali), *Nuzhenzi* (Fructus Ligustri lucidi), *Guizhi* (Ramulus Cinnamomi), *Chishao* (Radix Paeoniae rubra), *Huangqin* (Radix Scutellariae), *Huanglian* (Rhizoma Coptidis), *Shuizhi* (Hirudo)	Tonify qi and enrich yin, detoxify and resolve stasis.	*Huangqi* tonifies qi and strengthens defence-exterior; *guizhi* warms the channels and free yang; *nuzhenzi* nourishes yin of liver and kidney; *chishao* dissipates stasis and relieves pain; *huangqin* and *huanglian* clear heat, dry dampness and detoxify; *shuizhi* breaks blood and expels stasis.

Zeng (2005) [19]	Xiaokelingnongsuoye (Diabetes Agility Concentration Fluid)	Empirical formula	DPN, pattern of deficiency of qi and yin and blood stasis	*Sangshen* (Fructus Mori), *Shengdihuang* (Radix Rehmanniae), *Gegen* (Radix Puerariae), *Huangqi (*Radix Astragali), *Renshen* (Radix Ginseng), *Xuanshen* (Radix Scrophulariae), *Shuizhi* (Hirudo)	Nourish kidney yin, tonify qi and activate blood.	Chief: *sangshen* and *shengdihuang* nourish yin and tonify kidney. Deputy: *xuanshen* and *gegen* nourish yin and engender fluid. Assistant: *huangqi* and *renshen*, tonify qi; *shuizhi* is assistant, activate blood and remove stasis.

Xu (2009) [20]	Qingyingtang (Clearing Nutritive Qi Decoction)	From Wenbingtiaobian (Systematized Identification of Warm Pathogen Diseases)	Heat in the nutient aspect pattern, exhibiting fever which is higher at night, occasional delirium, restlessness, insomnia, thirst or no thirst, skin rashes, dry tounge and fine rapid pulse.	*Shuiniujiao* (Cornu Bubali), *Shengdihuang* (Radix Rehmanniae), *Xuanshen* (Radix Scrophulariae), *Zhuye* (Folium Phyllostachys nigrae), *Maidong* (Radix Ophiopogonis), *Danshen* (Radix Salviae miltiorrhizae), *Huanglian* (Rhizoma Coptidis), *Jinyinhua* (Flos Lonicerae), *Lianqiao* (*Fructus* Forsythiae)	Nourish yin and dispel heat and activate blood and free the channels.	Chief: *shuiniujiao* and *shengdihua* clear the heat in nutrient aspect and cool the blood. Deputy: *xuanshen* and *maidong* nourish yin and clear heat. Assistant: *jinyinhua, lianqiao, huanglian* and *zhuye* clear heat and detoxify, promote to dispel the pathogen from nutrient aspect to qi aspect; *danshen* activates blood and dispel stasis and heat.

Wang (2010) [21]	Tangmoning (Glucose End Peace Granules)	Empirical formula	DPN, pattern of qi deficiency and blood stasis	*Huangqi* (Radix Astragali), *Yanhusuo* (Rhizoma Corydalis), *Sanqi* (Radix Notoginseng), *Chishao* (Radix Paeoniae rubra), *Danshen* (Radix Salviae miltiorrhizae), *Chuanxiong* (Rhizoma Chuanxiong), *Honghua* (Flos Carthami), *Sumu* (Lignum Sappan), *Jixueteng* (Caulis Spatholobi)	Tonify qi, activate blood, free collaterals and relieve pain.	Chief: *huangqi* tonifies qi and promotes blood circulation. Deputy: *yanhusuo* and *chuanxiong* remove stasis and regender fresh blood. Assistant: *sanqi* removes stasis and relieve pain; *danshen, honghua* and *jixueteng* remove stasis and free collaterals.

Zhang (2008) [22]	Xiaoketongbi (Diabetes Free Obstruction Granula)	Empirical formula of Dr. Rongfa Zhang	DPN, pattern of deficiency of qi and yin and blood stasis	*Huangqi* (Radix Astragali), *Danggui* (Radix Angelicae sinensis), *Gegen* (Radix Puerariae), *Weilingxian* (Radix Clematidis), *Zushima* (Cortex Daphne giraldii), *Sangbaipi* (Cortex Mori)	Tonify qi, activate blood and remove stasis, free channels and relieve pain.	Chief: *huangqi, danggui* and *gegen* tonify qi, activate blood and relieve pain. Deputy: *weilingxian* and *zushima* dispel wind and relieve pain. Assistant: *sangbaipi* clears lung heat.

^∗^from Jinguiyaolue (Synopsis of Prescriptions of the Golden Chamber), which ingredients are *huangqi* (Radix Astragali), *guizhi* (Ramulus Cinnamomi), *shaoyao* (Radix Paeoniae), *shengjiang* (Rhizoma Zingiberis recens) and *dazao* (Fructus Jujubae).
